# Policy disparities in fighting COVID-19 among Japan, Italy, Singapore and China

**DOI:** 10.1186/s12939-020-01374-2

**Published:** 2021-01-13

**Authors:** Xiaohan Wang, Leiyu Shi, Yuyao Zhang, Haiqian Chen, Gang Sun

**Affiliations:** 1 Department of Health Management, School of Health Management, Southern Medical University, Guangzhou, Guangdong 510515 P.R. China; 2 Department of Health Policy and Management, Bloomberg School of Public Health, Johns Hopkins University, Baltimore, MD 21205 USA

**Keywords:** Global health equity, COVID-19, Pandemic response, Blocking measures, Mitigation measures

## Abstract

**Objective:**

In order to provide experiences for international epidemic control, this study systematically summarized the Coronavirus disease 2019 (COVID-19) prevention and control policies in Japan, Italy, China and Singapore, and also analyzed the possible inequalities that exist in these response approaches to improve global infectious disease control.

**Methods:**

We summarized the epidemic prevention and control policies in Japan, Italy, China, and Singapore, and analyzed the policy effects of these four countries by using the data published by Johns Hopkins Coronavirus Resource Center.

**Results:**

As of May 27, 2020, the growing trend of new cases in Japan, Italy, China and Singapore has stabilized. However, the cumulative number of confirmed cases (231139) and case-fatality rate (14.3%) in Italy far exceeded those in the other three countries, and the effect of epidemic control was inferior. Singapore began to experience a domestic resurgence after April 5, with a cumulative number of confirmed cases reaching 32,876, but the case-fatality rate remained extremely low (0.1%). The growth of cumulative confirmed cases in China (84547) was almost stagnant, and the case-fatality rate was low (5.5%). The growth of cumulative confirmed cases in Japan (16661) increased slowly, and the case-fatality rate (4.8%) was slightly lower than that in China.

**Conclusion:**

This study divided the epidemic prevention and control policies of the four countries into two categories: the blocking measures adopted by China and Singapore, and the mitigation measures adopted by Japan and Italy. According to the Epidemic control results of these four countries, we can conclude that the blocking measures were generally effective. As the core strategy of blocking measures, admitting mild patients into hospital and cases tracing helped curb the spread of the outbreak in Singapore and China. Countries should choose appropriate response strategies on the premise of considering their own situation, increase investment in health resources to ensure global health equity, and eventually control the spread of infectious diseases in the world effectively.

## Introduction

In late December 2019, the Coronavirus disease 2019 (COVID-19) emerged in the city of Wuhan, Hubei Province, China [1]. Using real-time reverse transcription polymerase chain reaction (RT-PCR), researchers identified the causative agent labeled as Severe Acute Respiratory Syndrome Coronavirus 2 (SARS-CoV-2) [2]. On 11 March 2020, the World Health Organization (WHO) announced that COVID-19 should be characterized as pandemic [3]. As of 27 May 2020, there have been 5,660,180 confirmed cases and 350,000 deaths reported worldwide [4]. The outbreak of COVID-19 pneumonia has produced high hospitalization rates after infection and an elevated mortality rate among the elderly aged over 60 [5]. It also has posed remarkable perils to the global health system, politics and economy. With no vaccine and no proven effective treatment, there is a compelling need to exert public health interventions to dominate its expansion [6].

Different interventions have been put in place by various countries to slow the spread of COVID-19 according to their national conditions. Some countries have applied rigorous blockade measures such as locking down schools, strict contact tracing and large-scale quarantine. China and South Korea are typical representatives of such countries. Many other countries enacted policies on travel restrictions and encouraged the limitation of social contacts, postponed events. These countries have taken relatively lenient measures to slow down the spread of the infection, trying to “flatten the curve” to prevent overwhelming health care systems. The United States, Britain, France, Japan tended to adopt such mitigation measures.

In this study, we chose China, a typical country with rigorous measures, and Japan, a typical country with mitigation measures respectively. Singapore seems to have remained relative normalcy of day-to-day life without taking strict blocking measures at the outbreak. However, the Singapore government has implemented early detection of cases through surveillance and aggressive contact tracing. Although Italy has imposed lockdown nationwide and added hard restrictions on commercial activities, it does not attach importance to case tracing and isolation of suspected cases. Then, we selected the two controversial countries and further studied the prevention and control policies adopted by these four countries in combination with China and Japan.

Based on the epidemic data of the four countries, we analyzed the epidemic control effect, explored the core of the epidemic control measures in the four countries, and provided practical experiences for international epidemic control. Inequity is a driving force in this pandemic. It is well known that many countries have underfunded health systems and precarious economies, so we also consider the possible inequalities between countries [7]. The research results would help policymakers to explore appropriate response strategies within the capabilities of each jurisdiction.

### Approaches of epidemic control in Japan, Italy, China, and Singapore

#### Japan’s approach

As shown in Table 1, the overall epidemic response in Japan was mainly divided into three phases. Phase 1 of Japan’s response was to prevent cases from being imported into Japan at an early stage when the virus had not yet spread in community. Japanese authorities focused on border control measures and issued outbreak risk warnings to the public. Phase 2 was marked by the “Basic Policy on COVID-19 Countermeasures” issued on 26 February 2020. The basic policy also reflected Japan’s overall epidemic precautionary ideas [8]. On the domestic confirmed case, Japan implemented a strategy of admitting severe cases into hospital and asking mild cases to stay in isolation at home. Japan also planned to raise SARS-CoV-2 RT-PCR testing standards and reduce health observations of close contacts. The Prime Minister also called on the public to exercise “self-restraint” and close down schools in phase 2. Concert and stage play across the country were suspended or postponed. Phase 3 focused on practicing social distancing. The Prime Minister issued an emergency declaration on 7 April 2020, requiring citizens to avoid unnecessary non-urgent outings, and all regions could expropriate pharmaceuticals. Citizens were asked to limit their contacts with other individuals and obey social distancing policies. But the emergency declaration was a request, not a mandatory one. It mainly depended on the consciousness and self-discipline of the Japanese nationals. The emergency declaration marked the beginning of a new stage of national epidemic prevention in Japan.

**Table 1:** Japan’s COVID-19 epidemic prevention and control policies

Phase	Policy	The Key elements
Phase 1	Issue risk warnings to the public	In response to changes in the epidemic situation of COVID-19 in Wuhan, Japanese Ministry of Foreign Affairs gradually raised the risk alert for the epidemic level. (1) On January 21, 2020, risk warning of infectious disease level 1 was issued throughout China. (2) On January 23, risk warning of infectious disease level 2 was issued against Wuhan, China. (3) On January 24, risk warning of infectious disease level 3 was issued for the whole of Hubei Province, including Wuhan, and recommended that travel in Hubei Province of China be suspended. (4) On March 18, risk warning of infectious disease level 1 was issued against globally.
	Border control measures	(1) On January 28, 2020, the Japanese Cabinet Meeting decided to designate COVID-19 as “designated infectious diseases” based on the “Infectious Disease Law”. Infected persons are prohibited from entering Japan. On February 1, the decree was implemented ahead of time. Relevant departments may require suspected patients to undergo examinations and be admitted to the hospital, prohibiting travelers from China’s Hubei and Zhejiang provinces from entering China. (2) Since then, the border control measures have been continuously upgraded. As of April 3, Japan has imposed entry restrictions on visitors from 73 countries and regions. Returning residents and long-term pass holders with travel history to these affected regions is subject to 14-day quarantine.
Phase 2	The ministry of health released the “Basic Policy on COVID-19 Countermeasures”	(1) The policy recommends that the public avoid gatherings, wash hands frequently, and observe cough etiquette. It is recommended that enterprises staggered commute and suspend school. (2) Unless the elderly and patients with underlying diseases, mild patients should in principle rest at home. If patients’ symptoms progress, then contact a medical institution for consultation. (3) Planning to change the standard of nucleic acid testing: at present, the testing standard is that doctors in various medical institutions judge whether to carry out testing. If patient’s number continues to increase in the future, it will be changed to test pneumonia patients who need to be admitted to hospital. (4) Planning to reduce the observation of close contacts: at present, Japan conducts an epidemiological survey of close contacts of confirmed patients. However, if patient’s number continues to increase in the future, it will be changed to “reduce the health observation of close contacts”.
	The Prime Minister called on the public to “self-restraint”	On February 26th, the Japanese Prime Minister called for large-scale cultural and sports activities to “self-restraint” for 2 weeks. Therefore, concerts and stage play across the country were suspended or postponed. Tokyo Disneyland and Universal Studios also announced temporary closures. March 10 Japan has added the requirement of “self-restraint for 10 Days”.
	The Prime Minister called on school closures	The prime minister called on primary and secondary schools across the country to suspend classes from March 2 until spring break.
Phase 3	Declaring a state of emergency	The prime minister declared a state of emergency on 7 April 2020, encouraging people to avoid unnecessary outings and to observe social distancing. On May 25, Japan lifted the declaration of emergency.

Health expenditure per capita in Japan in 2017 was USD$4168.99 [9]. Japan has 13.7 beds per thousand people, which is much higher than the world average of 3.7 beds [10]. Per capita medical resources are relatively abundant in Japan. However, the capacity of infectious diseases is limited, because the beds of infectious diseases are mainly concentrated in public hospitals, with a relatively small ratio. At a press conference on 29 February 2020, the Prime Minister proposed to invest 270 billion Yen in the anti-epidemic reserve, and ensured that there are 5000 beds for infectious disease patients in the country [11]. Additionally, Japan ranked first in the world in terms of aging degree in 2019, with 28% of the population over 65 years old. Japan experiences severe risks in this context [12].

#### Italy’s approach

As shown in Table 2, Italy’s overall response to the epidemic was mainly divided into three phases. Phase 1 focused on border control prevention. Italy cut off all flights with China as early as 31 January 2020, and set up specialized agency at the national level to promote the response to the epidemic. At the same time, the Italian government established a surveillance system for COVID-19, which attached importance to the SARS-CoV-2 RT-PCR testing of suspected cases.

**Table 2:** Italy’s COVID-19 epidemic prevention and control policies

Phase	Policy	The Key elements
Phase 1	Set up a special agency to promote the epidemic response	On 22 January, 2020, the Working Group on Response to COVID-19, led by the Italian Minister of Health, was established to discuss the progress of the epidemic and propose measures to prevent and control the epidemic.
	Border control measures	(1) On January 31, 2020, the Italian Prime Minister announced that the country had entered a state of emergency for 6 months, cutting off all flights to and from China. (2) On February 3, the Ministry of Foreign Affairs sent military aircraft to bring Italian citizens in Wuhan back home, and conducted 14-day quarantine. (3) On February 22, the Prime Minister announced that all passengers who had traveled to China 14 days prior to entry must accept isolation at home or hotel. Close contacts must be forced to isolate for 14 days. (4) From March 13 to March 16, several terminals were closed, and many civil flights at major airports and international flights from Milan were grounded.
Phase 2	Public health response	(1) On February 22, 2020, Italy imposed a lockdown with shutdown of businesses, schools and public places plus physical distancing in Milan and Venice. (2) On March 1, the whole country of Italy was divided into red zone, yellow zone and safe zone. In addition to the “isolated quarantine” of the red zone, yellow zone suspended social and sports activities and closed Schools, clubs, theaters and cinemas. (3) The precautionary measures continue to be upgraded. On March 10, Italy imposed a lockdown with prohibiting all public gatherings and suspending all sports events in the whole country. (4) On March 11, Italy required all commercial activities to be stopped except for pharmacies and supermarkets that supply essential necessities throughout the country. (5) On March 20, the Italian government added restrictions on public travel and commercial activities. The next day, all non-essential production activities were stopped nationwide. Remote office should be implemented in non-essential situations. The Prime Minister announced the implementation of this series of prevention and control measures until May 3, and entered ongoing epidemic prevention and control phase.
	More stringent testing policies	(1) On February 25, 2020, the Italian Ministry of Health issued more stringent testing policy. This recommendation prioritized testing for patients with more severe clinical symptoms. (2) On February 27, the Italian Ministry of Health announced that it would revise the current method of accounting for confirmed cases in accordance with the standards of WHO and the European Center for Disease Control: Asymptomatic positive patients and people who have not undergone secondary tests will not be included in the confirmed data.

On 20 February 2020, a severe case of COVID-19 was diagnosed in northern Italy’s Lombardy region in a man (patient 1) who had no history of possible exposure abroad. During the next 24 h, 36 additional cases were confirmed, without being linked to patient 1 or previously identified positive cases already in the country [13]. Phase 2 was to take rigorous public health measures with the advent of patient 1. Italy divided the country into Red Zone, Yellow Zone or Safe Zone. The government took different precautions according to different zones to prevent the spread of the epidemic. On 10 March 2020, Italy imposed a lockdown with prohibiting all public gatherings and suspending all sports events in the whole country. However, whether in the Red Zone, Yellow Zone or Safe Zone, Italy only attached great importance to treating severe cases, and does not accept hospitalization for mild patients. The authorities required that patients who are asymptomatic or have mild symptoms would be asked to stay in isolation at home. The Italian Ministry of Health also issued more stringent testing policies on 25 February 2020. Testing was limited for asymptomatic people or people with mild symptoms [14]. In phase 3, the precautionary measures were gradually relaxed after May 3, and the stage of ongoing epidemic prevention and control was entered.

Apparently, the Italian health systems have not equipped in time to deal with this pandemic. With the large-scale reduction of public hospitals, Italy’s healthcare system has undergone some important changes since 2000. Furthermore, the accessibility and functionality of local health services are extremely different among regions [15]. Health expenditure per capita in Italy in 2017 was USD$2840.13 [9]. There were only 6000 intensive care units in Italy early in the outbreak [16]. In addition, as of January 2019, Italy had 13.89 million people over the age of 65, accounting for 22.8% of the total population. Italy ranks second in the world in terms of aging, after Japan [12].

#### China’s approach

In late December 2019, COVID-19 outbreak in Wuhan, Hubei Province, China. As the first country hit by COVID-19, China initiated an extraordinary community containment effort in history. Table 3 shows the main epidemic prevention and control policies adopted by China. Marked by locking down Wuhan on 23 January 2020, China launched an unprecedented large-scale public health measure. It was a unique feature of the Chinese political system to establish a wartime working mechanism led by the central government and mobilize the whole country to fight the epidemic. China’s core measures are to strictly observe the principle of early cases detection, reporting, quarantine and treatment, and to put four categories of people – confirmed cases, suspected cases, febrile patients in whom COVID-19 could not be excluded, and close contacts – under classified management in designated facilities. These two measures effectively isolated the source of infection and cut off the route of transmission while preventing cross-infection. China had made every effort to track down, isolate, and treat COVID-19 patients to curb the spread of the virus nationwide. On April 7, 2020, Wuhan lifted lockdown, and nationwide epidemic prevention and control was being conducted on an ongoing basis.

**Table 3:** China’s COVID-19 epidemic prevention and control policies

SN	Policy	The Key elements
1	Lockdown Wuhan	On January 23, 2020, the government put the city under lockdown by shutting services at the airport, railway stations, ferry ports and long-distance bus stations. On April 8, Wuhan lifted lockdown.
2	Establishing a Leading Group to combat COVID-19	(1) The central government established a leading group for epidemic response work. (2) The central government dispatched guidance groups to Hubei and other epidemic-stricken areas to uniformly guide local epidemic control.
3	The principle of early cases detection, reporting, quarantine and treatment	(1) On February 3, President Xi required that epidemic control measures be improved and strengthened and that the principle of early detection, reporting, quarantine and treatment be strictly observed. (2) He called for saving lives by raising admission and cure rates and lowering infection and fatality rates.
4	Classified management of “four categories of personnel”	(1) Wuhan began to adopt measures to put four categories of people – confirmed cases, suspected cases, febrile patients who might be carriers, and close contacts – under classified management in designated facilities. The policy of ensuring that all those in need are tested, isolated, hospitalized or treated was implemented. (2) Actions were taken to conduct mass screenings to identify people with infections, hospitalize them, and collect accurate data on case numbers in the whole country.
5	Counterpart assistance	(1) Mobilizing national medical resources to fully support medical treatment in Hubei Province and Wuhan City. From January 24 to March 8, a total of 346 national medical teams, 42,600 medical personnel and more than 900public health workers were mobilized to assist Hubei. (2) Establishing an inter-provincial counterpart support mechanism for COVID-19 medical treatment in cities other than Wuhan in Hubei Province. (3) Mobilizing 40,000 builders and thousands of mechanical equipment from all over the country, built the Huoshen Shan Hospital with 1000 beds in only 10 days, and built the Leishenshan Hospital with 1600 beds in only 12 days. In just over 10 days, 16 mobile cabin hospitals were built, with a total of more than 14,000 beds. (4) The central government cooperated with local governments and enterprises to supply living materials in Hubei Province and Wuhan City to ensure the normal operation of the society.
6	Nationwide public health measures	(1) Temperature screening is set up in various places across the country. (2) Taking effective measures to avoid personnel gathering and cross-infection: extend the Spring Festival holiday, cancel or postpone gathering activities, lock down various schools; close entertainment venues; public service places that need to be opened must take body temperature and wear Masks; encourage employees to telecommute. (3) Implementing community closed management nationwide. Residents in and out of the community register and check their body temperature. (4) Carrying out extensive public education in community. Residents consciously implement public health requirements such as home isolation and 14 days after cross-regional travel, strictly implement health living habits such as wearing masks, observing social distance, reducing gathering.

China’s per capita health resources are inadequate, with only 3.4 beds per thousand people [10]. Health expenditure per capita in China in 2017 was only USD$440.83 [9]. However, the Chinese government has restored the collapse of health services system in Hubei Province by adopting “pairing assistance” nationwide [17]. China concentrated all the people’s efforts to solve the spatial inequality of health resources in Hubei Province. A total of 346 medical teams composed of 42,600 medical workers and 965 public health workers from across the country and the armed forces were dispatched to Hubei and Wuhan [18].

#### Singapore’s approach

As shown in Table 4, the overall epidemic response in Singapore was mainly divided into three phases. Phase 1 focused on border control prevention to prevent inbound cases. The Singapore government rapidly set up a Multi-Ministry Task Force and attached importance to the isolation of early-detected cases and close contacts. The government identified Wuhan-related personnel by the primary health care self-government system. With the advent of local human-to-human cases in Singapore on 4 February 2020, the number of confirmed cases in Singapore had been gradually increasing. Phase 2 focused on ongoing community and social measures. Relative normalcy of day-to-day life had been maintained in Singapore. The Singapore government established a strict case surveillance system, and SARS-CoV-2 RT-PCR laboratory testing was scaled up rapidly to all public hospitals [19]. Accompanied by the mature primary care setting, the Singaporean government implemented hierarchical diagnosis in the country to ensure that the treatment of mild and severe patients while preventing cross-infection. Phase 3 was to prevent domestic resurgence in infections as the number of foreign labor dormitory cases had increased significantly [20]. The Singapore government has tightened a series of epidemic prevention and control measures since 7 April 2020, implementing school closures and other major social-distancing measures to contain resurgence.

**Table 4:** Singapore’s COVID-19 epidemic prevention and control policies

Phase	Policy	The Key elements
Phase 1	Setting up a Multi-Ministry Task Force	The preliminary plan of the Multi-Ministry Task Force, drawn up after the 2003 SARS outbreak, was launched on 22 January 2020 to coordinate among departments and provide strategic and political guidance during the public health crisis.
	Early isolation and early screening	(1) On January 23,2020, Singapore set up a special team with the help of the information-based public health system to thoroughly investigate the personnel related to Wuhan. (2) Isolation of early cases: On January 26, Singapore confirmed four cases of COVID-19. The Singapore government expropriated some student dormitories as isolation facilities. (3) The government had issued a law on home quarantine: from February 18, Singapore tightened the isolation regulations related to COVID-19 and issued a legally binding “stay at home notice”. Those who receive the notice shall not go out during home quarantine, otherwise they may face heavy penalty.
	Border control measures	(1) Temperature and health screening of incoming travellers from Wuhan since January 3, 2020, and extended to all travelers since January 29, 2020, is in place at all ports of entry. Travellers who meet the suspect case definition are conveyed directly to hospital. (2) Singapore imposed entry restrictions on visitors from countries in outbreaks such as China, ROK, Northern Italy and Iran. (3) From March 5, all inbound passengers who have symptoms such as fever and cough will be required to undergo a throat swab sample test. (4) From March 23, short-term visitors and cruise ships are prohibited from docking.
Phase 2	Surveillance measures	(1) According to the time and distance of contact with the confirmed cases, the contacts are divided into two categories, and tracking are carried out separately. Close contacts will be forced to be isolated for 14 days, and low-risk contacts will be actively detected. (2) On March 21, 2020, the government launched the “Trace Together” APP for tracking close contacts of confirmed cases.
	Community and social measures	(1) The government only encouraged ill persons to wear masks to prevent them from infecting other. On February 1, 2020, the government distributed masks free of charge to residents across the country, with four masks per family. (2) On February 4, employees were encouraged to monitor their temperature and health regularly in workplace. (3) The school remains open, but implemented preventive measures, such as reducing group meetings and staggering meal times. (4) With the escalation of the epidemic situation, the taxi service will be stopped on February 9 and all activities with more than 50 people will be cancelled. Necessary activities must be recorded and turn away ill individuals.
	Mature primary care setting	(1) Majority of cases were isolated and treated at the National Centre for Infectious Diseases (NCID), a 330-bed purpose built infectious diseases management facility. NCID can accommodate nearly 500 beds during an outbreak, enhancing Singapore’s infectious disease prevention capabilities. (2) A network of > 800 Public Health Preparedness Clinics (PHPCs) was activated to enhance management of respiratory infections in the primary care setting, with subsidies extended to Singapore residents to incentivize them to seek care at these PHPCs. If it is highly suspected to be COVID-19, refer to the general hospital.
Phase 3	Strict community-wide measures	(1) Since April 5, 2020, the Singapore government has distributed issued reusable masks to every household. At the same time, regardless of whether they wear masks, the government recommended that everyone wash their hands and observe social distance. (2) From April7, all workplaces and shops providing non-essential services will be closed; (3) From April 8, schools and pre-school education institutions will be closed and changed to home study; (4) The public should stay at home as much as possible and not go out as much as possible. The gathering is limited to family members living together. (5) Enterprise employees must work at home.

Health expenditure per capita in Singapore in 2017 was USD$2618.71, and the number of beds per thousand people was 2.4, which was not rich compared with Japan [9, 10]. But Singapore has steadily built up its infectious disease preparedness since the 2003 SARS outbreak. Infrastructure for outbreak management was significantly augmented. The National Centre for Infectious Diseases (NCID), a 330-bed purpose-built infectious disease management facility was launched quickly. A network of > 800 Public Health Preparedness Clinics (PHPCs) was activated to enhance management of respiratory infections in the primary care setting [21]. Early action helped Singapore upgrade its medical capabilities to cope with the surge in patients.

### Epidemiological trends of COVID-19 in four countries

Data on COVID-19 cases in Japan, Italy, China and Singapore were obtained from the website of Johns Hopkins Coronavirus Resource Center. These publicly available data were collated and analyzed for the effectiveness of the control measures implemented in the four countries. The efficacy of these measures implemented between 23 January 2020 and 27 May 2020 was evaluated, with a focus on the trend of the cumulative confirmed cases and the case-fatality rate.

**Fig. 1:**
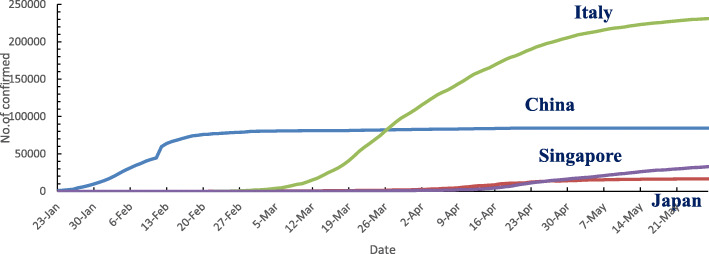
Cumulative confirmed case trends in four countries

As seen in Fig. 1, the cumulative confirmed cases in Japan, Italy, China, and Singapore showed different trends from 23 January to 27 May. In China, such cases had been declining since mid-February and then had been maintained at a stable state for a long time. In Japan, such cases increased slowly in the early stage of Phases 1 and 2 and then rapidly from late April to early May. Since late May, the cumulative growth rate of the confirmed cases had slowed down, and then the epidemic situation there gradually went stable. In Singapore, such cases did not exceed 1000 before April 1. Since April 5, Singapore had experienced domestic resurgence in infections, with the number of the confirmed cases increasing rapidly and the cumulative number of confirmed cases gradually exceeding that of Japan. In Italy, although the epidemic emerged relatively late, the number of confirmed cases continued to grow rapidly until April 30, after which, the growth rate slowed down, showing a trend of alleviation.

**Fig. 2:**
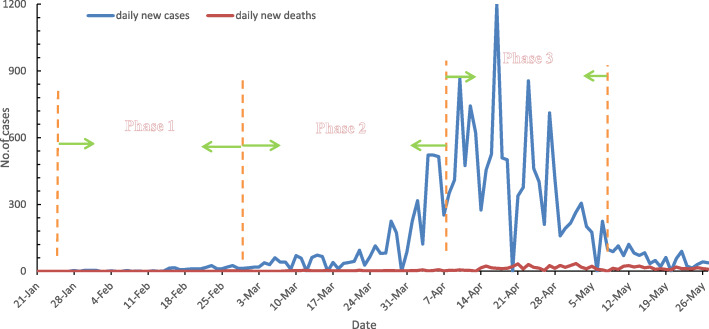
Trends of daily new cases and new deaths in Japan

**Fig. 3:**
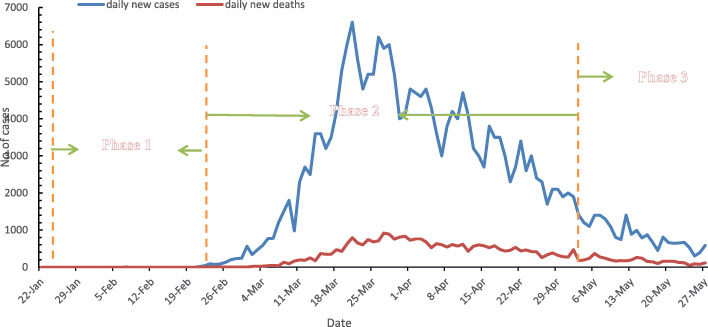
Trends of daily new cases and new deaths in Italy

**Fig. 4:**
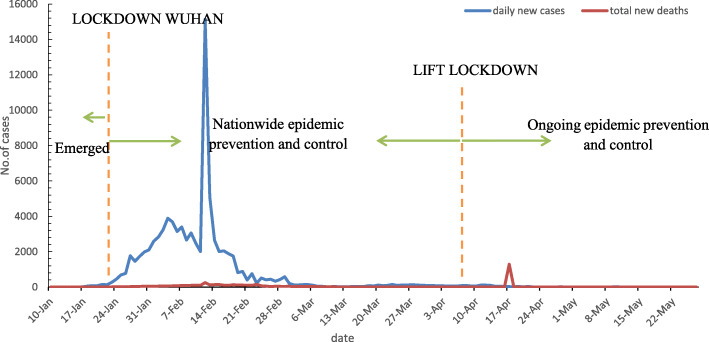
Trends of daily new cases and new deaths in China

**Fig. 5:**
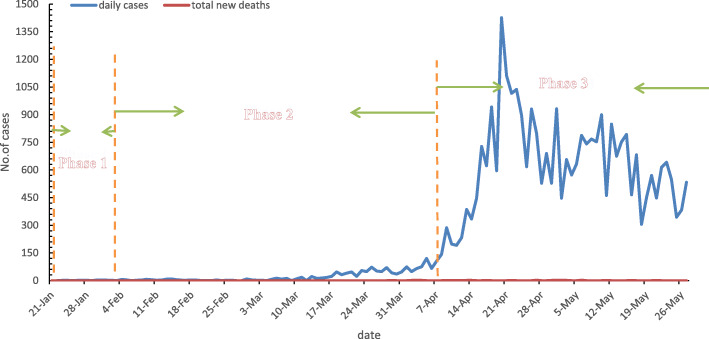
Trends of daily new cases and new deaths in Singapore

Figures 2, 3, 4 and 5 show the trend of daily new cases and new deaths as well as the prevention and control phases since the emergence of COVID-19 in the four countries, respectively. The number of new deaths in Japan from Phases 1 to 3 remained at a relatively low level. The curve of daily new cases showed an upward trend with fluctuations, but the number of new cases dropped significantly in the late period of Phase 3. The overall trend of new deaths in Italy was similar to that of its daily new cases: The curve rose rapidly in the early period of Phase 2, slowed down gradually in the later period, and then entered a steady trend in Phase 3. Compared with other countries, Italy has experienced a larger number of deaths and confirmed cases. China was fast in controlling the epidemic and had kept the number of new cases and deaths at a low level and maintained a steady trend for a long time in the early period of nationwide lockdown. On the other hand, in Singapore, the number of newly confirmed cases was relatively small in Phases 1 and 2, with a low growth rate. Then in the early period of Phase 3, the number of newly confirmed cases increased rapidly, but a downward trend was showed in the late Phase 3, while the number of new deaths had always remained extremely low from Phase1 to Phase3.

**Fig. 6:**
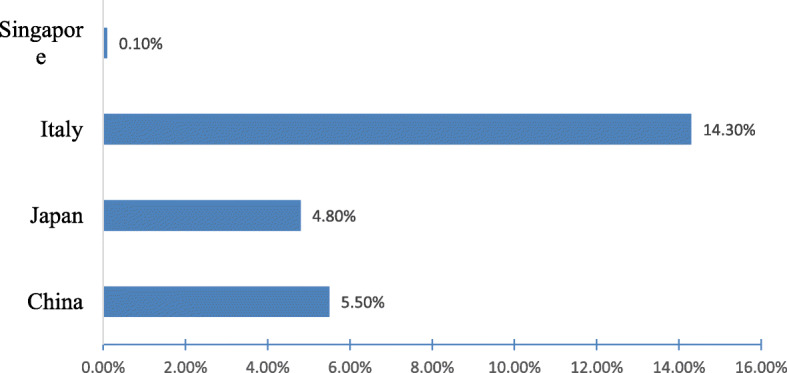
Comparison of case-fatality rates (as of May 27) in four countries

Figure 6 shows the case-fatality rate (deaths/confirmed cases) of Japan, Italy, China and Singapore as of 27 May 2020. The overall case-fatality rate in Italy (14.3%) is substantially higher than that in Japan, China and Singapore, with the rate in Japan (4.5%) slightly lower than that in China (5.5%). And that in Singapore shows an extremely low value of 0.1%.

## Discussion

There are differences in the epidemic situations, medical systems, precautionary principles and cultural customs in Japan, Italy, China and Singapore, so the prevention and control policies adopted against the COVID-19 epidemic varied. Based on the nature of interventions, this study classifies the epidemic prevention and control policies of the four countries into two categories: the blocking measures taken by China and Singapore, and the mitigation measures taken by Japan and Italy.

COVID-19 affects all people in the era of globalization. As “Buckets Effect” reveals: the capacity of a bucket depends on the shortest wooden board [22]. The successful control of the global epidemic depends on the worst-case countries. To play the overall role of the global health system, we should not only to play its advantages, but also make up for its deficiencies. As we all known that many countries have underfunded health systems and precarious economies. Inequity is a driving force in this pandemic both between countries and within countries. For our health and the health of the world, we also focus on the possible inequalities in these countermeasures, so as to help policy makers and governments to make appropriate responses and eventually overcome COVID-19.

### Blocking measures

China, which adopted blocking measures, attached more importance to the implementation of more aggressive closed management measures. After the outbreak, the government decisively decided to lock down the metropolitan city to contain the spread of the infectious virus to other regions, while implementing large-scale precautions nationwide, including school closure, work suspension, and production stoppage, and community containment, purposed to restrict national activities [23].

These radical closure management measures promoted the core measures to play a better control role. The core of the blocking measures was to treat mild patients and track down cases. The Chinese government performed case hospitalization and isolation through nationwide screening of confirmed patients, suspected patients, febrile patients and close contacts. Heavy efforts were made to ensure that all patients with COVID-19 pneumonia were admitted into special hospitals and all relevant persons were screened. In this way, the sources of infection were effectively isolated and the transmission routes were cut off.

In Singapore, although its aggressive community-wide measures were only taken after the resurgence of infections, the government was focused much on extensive testing and case tracing in Phases 1 and 2. The government established a strict case surveillance system, and laboratories were enhanced for their SARS-CoV-2 RT-PCR testing capabilities. The primary healthcare system also cooperated with the implementation of case surveillance. Further, the government issued a law on home quarantine to ensure that the specified persons strictly stayed at home. Furthermore, by virtue of its mature primary public health system, Singapore implemented a hierarchical diagnosis system during the outbreak, so as to decentralize treatments of mild and severe cases and alleviate medical runs.

The epidemic prevention and control effect in China and Singapore show the implementation of blocking measures is effective. Through large-scale screening, COVID-19 cases can be fully discovered and isolated early, and mild and severe patients can be treated comprehensively. As a result, the case-fatality rate of COVID-19 can be effectively controlled, with the transmission of the novel coronavirus finally reduced.

### Mitigation measures

Mitigation measures can be taken to slow down the spread of COVID-19, so that it spreads slowly in a controlled state, with focus on the treatment of severe cases. Meanwhile, in order to avoid medical runs, social distancing measures shall be put in place when necessary. However, early detection of all cases and identification of close contacts are not the focus, and the treatment of mild patients is not in priority.

In Japan, an important feature of the mitigation measures was to implement the countermeasures that admitted severe cases into hospital and asked mild cases to stay at home. In addition, Japan also raised testing standards and reduced the health observations on close contacts. The goal was to slow down the spread of the Epidemic as much as possible and restrain the peak of the incidence curves, while ensuring that the health care system would not collapse due to excessive pressures and the domestic health losses be controlled to the minimum level. This goal’s realization required the efforts of all residents. The policies issued by the Japanese government for COVID-19 were mostly not mandatory and had no legal effect. However, thanks to the self-discipline and health literacy of Japanese residents, the outbreak has also entered a relatively stable trend after April in Japan, with a relatively low case-fatality rate (4.8%).

In Italy, although it had quickly imposed lockdown measures similar to China after the surge in domestic infections, it did not attach importance to the treatment of mild patients, but advocated the isolation of mild patients at home. The Italian Ministry of Health has issued certain more stringent testing policies, so as to only detect the high-risk groups with symptoms. The essence of the Italian precautionary policies is to take mitigation measures [24]. However, the changed Italian health system was unprepared to face a pandemic. With the increase of the total number of the infectious cases, a medical run occurred in Italy.

### Inequities behind response measures

There is no doubt that response COVID-19 by blocking measures needs to pay a heavy economic price. The completion of case tracing requires mass testing, and mass testing would require substantial investment in lab facilities, equipment and medical technique personnel. Considering the inequality of health expenditure between high-income countries and low-income countries, can low-income countries with relatively weak economic infrastructure provide the same scale of testing? [25] Hospitalization of mild and severe patients need a large number of beds and medical staff. It is also difficult for many countries to invest large amounts of health resources. Mitigation measures to prevent the collapse of the health care system seem to be optimal solution. However, the mitigation measures require the mild patients to be quarantined at home, which makes it difficult for people living in poor conditions to be adequately isolated from other family members, exacerbating the health risks of vulnerable groups in the country and exacerbates health inequalities [15].

Furthermore, COVID-19 disproportionately affected the already marginalized groups in global, such as the socio-economically disadvantaged and the elderly. Socio-economically disadvantaged often faces higher exposure risks during the pandemic. The most vulnerable age group is the elderly, especially those living in nursing homes, who account for almost half of deaths in COVID-19 [26]. Decision-makers should ensure the life safety and the right to health of marginalized groups, when comparing policy options for tackling the pandemic. Investment in health resources is a necessary way to ensure the accessibility and equality of health services [27]. High-income countries should increase the proportion of investment in health resources, while low-income countries should also seek opportunities for international health funding. No country can immune to this crisis, so we must strengthen collaboration and equity in the field of global health in order to win the eventual victory in fighting infectious diseases.

## Conclusion

This study finds that China and Singapore had a better control effect by implementing blocking measures, while Japan had achieved a better control effect by implementing mitigation measures and depending on its national self-discipline and good health literacy. In Italy, however, the implementation of mitigation measures did not attach importance to the admission of mild patients and case tracking; and coupled with the aging population, the number of confirmed cases and case-fatality remained high, with an inferior epidemic control effect. We can conclude that the blocking measures were generally effective. As the core strategy of blocking measures, admitting mild patients into hospitals and conducting cases tracking helped Singapore and China contain the spread of the outbreak. Countries should choose appropriate response strategies on the premise of considering their own situation, increase investment in health resources to ensure global health equity, and eventually control the spread of infectious diseases in the world effectively.

## Data Availability

The datasets analyzed during the current study are available in the Johns Hopkins Coronavirus Resource Center repository, [https://coronavirus.jhu.edu/].

## Abbreviations

COVID-19: The Coronavirus disease 2019
RT-PCR: real-time reverse transcription polymerase chain reaction
SARS-CoV-2: Severe Acute Respiratory Syndrome Coronavirus 2
WHO: The World Health Organization
